# The discovery of rivaroxaban: translating preclinical assessments into clinical practice

**DOI:** 10.3389/fphar.2013.00145

**Published:** 2013-11-25

**Authors:** Dagmar Kubitza, Elisabeth Perzborn, Scott D. Berkowitz

**Affiliations:** ^1^Clinical Pharmacology, Bayer HealthCare AGWuppertal, Germany; ^2^Formerly Global Drug Discovery, Bayer Pharma AGWuppertal, Germany; ^3^Bayer HealthCare PharmaceuticalsWhippany, NJ, USA

**Keywords:** anticoagulation, factor Xa inhibition, pharmacodynamics, prothrombin time, rivaroxaban, thrombin generation

## Abstract

Direct oral anticoagulants that target a single coagulation factor (such as factor Xa or thrombin) have been developed in recent years in an attempt to address some of the limitations of traditional anticoagulants. Rivaroxaban is an oral, direct factor Xa inhibitor that inhibits free and clot-bound factor Xa and factor Xa in the prothrombinase complex. Preclinical studies demonstrated a potent anticoagulant effect of rivaroxaban in plasma as well as the ability of this agent to prevent and treat venous and arterial thrombosis in animal models. These studies led to an extensive phase I clinical development program that investigated the pharmacological properties of rivaroxaban in humans. In these studies, rivaroxaban was shown to exhibit predictable pharmacokinetics and pharmacodynamics and to have no clinically relevant interactions with many commonly prescribed co-medications. The pharmacodynamic effects of rivaroxaban (for example, inhibition of factor Xa and prolongation of prothrombin time) were closely correlated with rivaroxaban concentrations in plasma. The encouraging findings from preclinical and early clinical studies were expanded upon in large, randomized phase III studies, which demonstrated the clinical efficacy and safety of rivaroxaban in a broad spectrum of patients. This article provides an overview of the discovery and development of rivaroxaban, describing the pharmacodynamic profile established in preclinical studies and the optimal translation to clinical studies in healthy subjects and patient populations.

## Introduction

Anticoagulant drugs are routinely used for the prevention and treatment of thromboembolic disorders. Although effective, traditional anticoagulant agents (which have a broad effect on multiple coagulation factors) are associated with several limitations. For example, heparins require a parenteral route of administration, and unfractionated heparin and vitamin K antagonists have significant variability in their pharmacodynamic responses, thus requiring routine coagulation monitoring and dose adjustments (McRae and Ginsberg, 2004; Ageno et al., 2012; Garcia et al., 2012). These shortcomings have spurred the search for novel agents that specifically target a single clotting factor within the coagulation cascade, such as factor Xa or thrombin.

**Figure 1:**
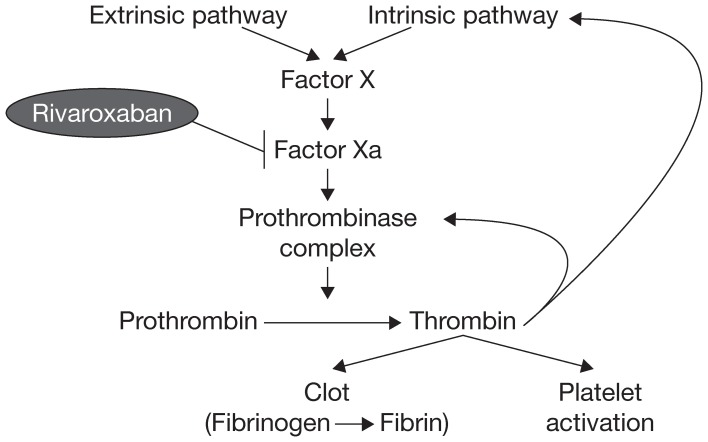
**The blood coagulation pathway**.

Factor X has long been known to have a key role in hemostasis, and its activated form, factor Xa, has a significant function in the blood coagulation pathway because it catalyzes the production of thrombin, which leads to clot formation (Koller, 1960; Leadley, 2001). Activation of factor X to factor Xa occurs through both the intrinsic and extrinsic pathways of the coagulation cascade. Factor Xa initiates the final, common pathway that results in thrombin activation via the prothrombinase complex (Figure 1). It has been estimated that one molecule of factor Xa can catalyze the production of ~1000 molecules of thrombin because of the amplification inherent in the coagulation cascade (Rand et al., 1996; Mann et al., 2003; Kubitza and Haas, 2006; Perzborn et al., 2011); therefore, effective thrombin inhibition requires sustained high levels of free inhibitor under conditions of thrombin “burst.” These requirements are expected to be less stringent for factor Xa, because factor X is present in plasma at much lower concentrations compared with prothrombin (Brummel-Ziedins et al., 2012). Factor Xa is, therefore, an attractive target for anticoagulant agents.

Early studies of naturally occurring factor Xa inhibitors indicated that targeting factor Xa could provide effective anticoagulation (Dunwiddie et al., 1989; Nicolini et al., 1996). Further studies with the synthetic indirect factor Xa inhibitor fondaparinux provided the proof of principle that selective inhibition of factor Xa could provide clinically effective anticoagulation (Turpie et al., 2002). Selective inhibition of factor Xa produces antithrombotic effects by decreasing the generation of thrombin, thus diminishing thrombin-mediated activation of both coagulation and platelets without affecting the activity of existing thrombin (Ieko et al., 2004).

Among the novel, direct, oral factor Xa inhibitors that have been developed in recent years, rivaroxaban was the first to gain regulatory approval for clinical use (which occurred in 2008). The pharmacodynamic effects of rivaroxaban were first shown in preclinical studies, and were subsequently demonstrated in early clinical studies in humans. The promising results from the early phase of development were confirmed in large-scale phase III studies, in which the efficacy and safety of rivaroxaban were demonstrated. Rivaroxaban is now approved for clinical use for the prevention and treatment of venous and arterial thromboembolic disorders (Bayer Pharma AG, 2013). This article will provide an overview of the discovery and development of rivaroxaban, describing the pharmacodynamic profile established in preclinical studies and the optimal translation to clinical studies in healthy subjects and patient populations.

## Preclinical pharmacodynamic profile

High-throughput screening of approximately 200,000 compounds revealed several hits that selectively inhibited the cleavage of a chromogenic substrate by human factor Xa. The subsequent optimization program led to the identification of rivaroxaban as the lead compound for further development because of its high binding affinity to factor Xa, potency *in vitro*, and *in vivo* antithrombotic activity, as well as its favorable oral bioavailability (Perzborn et al., 2011).

The mode of action of rivaroxaban is the direct and specific competitive inhibition of factor Xa (inhibition constant [K_*i*_] 0.4 ± 0.02 nM), with >10,000-fold selectivity for factor Xa over other serine proteases (Perzborn et al., 2005). Unlike the indirect factor Xa inhibitor fondaparinux, or low molecular weight heparins and unfractionated heparin, which exert their actions via antithrombin, rivaroxaban directly inhibits prothrombinase (half maximal inhibitory concentration [IC_50_] 2.1 ± 0.40 nM). In human plasma, rivaroxaban also inhibits the activity of clot-bound factor Xa (IC_50_ 75 nM) in a concentration-dependent manner (Depasse et al., 2005).

In whole blood and platelet-rich human plasma, nanomolar concentrations of rivaroxaban significantly prolonged the initiation phase of thrombin generation (TG), significantly reduced the rate of the propagation phase of coagulation after tissue factor (TF) activation, and led to a reduction in endogenous thrombin potential (ETP)—a measure of the activity of thrombin multiplied by the time for which it remains active (Gerotziafas et al., 2007). Rivaroxaban reduced the ETP by 50% at a concentration of 35 nM; TG was almost completely inhibited in platelet-rich plasma at physiologically relevant concentrations (80–100 nM) of rivaroxaban (Gerotziafas et al., 2007). In platelet-poor human plasma, rivaroxaban prolonged prothrombin time (PT; assessed using Neoplastin Plus® from Roche Diagnostics, Mannheim, Germany) in a concentration-dependent manner; the assay concentration required to double the PT was ~691 nM (Perzborn et al., 2005).

**Figure 2:**
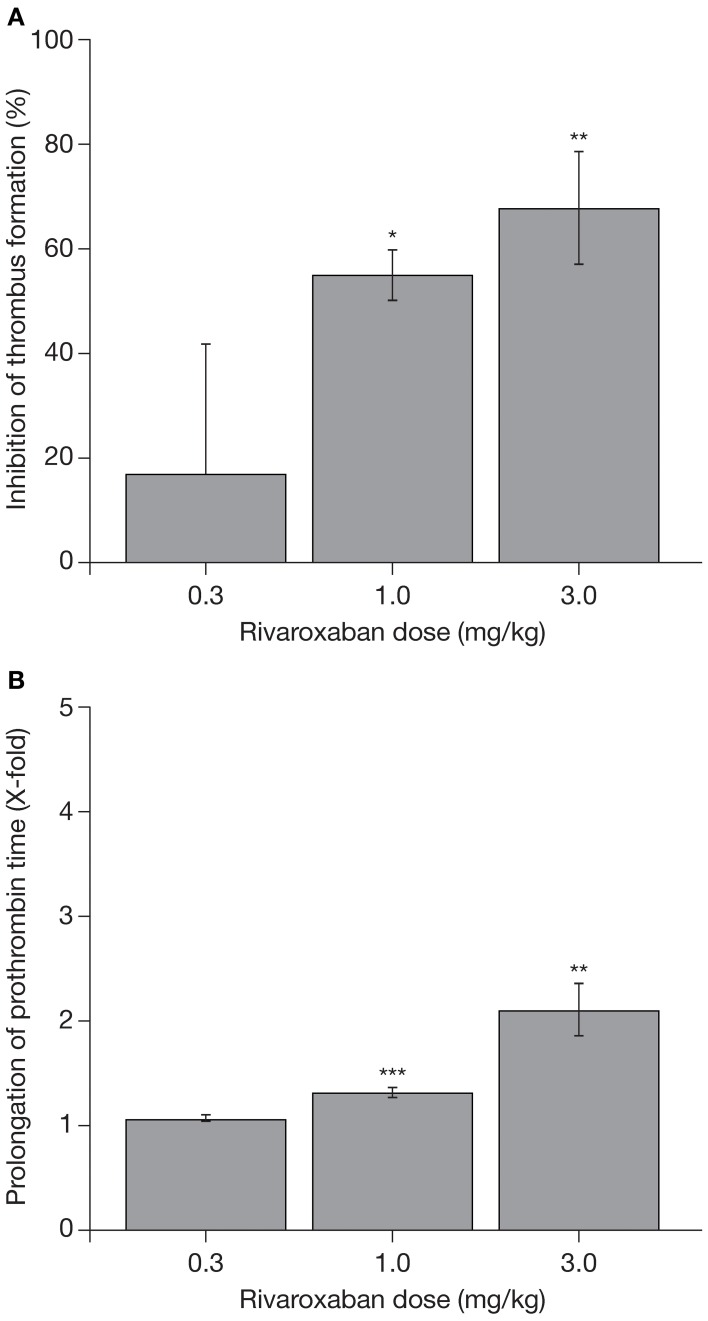
**(A)** Reduction in thrombus formation and **(B)** prolongation of prothrombin time with rivaroxaban in a rabbit arteriovenous shunt model. Each value represents the mean ± SEM of six animals. ^*^p < 0.05; ^**^p < 0.01; ^***^p < 0.001. Data published previously in the *Journal of Thrombosis and Haemostasis* (Perzborn et al., 2005).

The promising data on the pharmacodynamic effects of rivaroxaban obtained in these coagulation tests were supported by studies in animal models of venous and arterial thrombosis. In a rat venous stasis model, PT and factor Xa activity were affected slightly at the half maximal effective dose (ED_50_) of rivaroxaban (1.8-fold increase and 32% inhibition, respectively) and, at a dose leading to almost complete inhibition of thrombus formation (0.3 mg/kg), rivaroxaban prolonged PT moderately (3.2 ± 0.5-fold) and inhibited factor Xa activity (65 ± 3%) (Perzborn et al., 2005). In a rat arteriovenous shunt model, oral rivaroxaban inhibited factor Xa activity by 74% and prolonged PT by 3.2-fold; in a rabbit arteriovenous shunt model, factor Xa activity was almost completely inhibited (92%) by oral rivaroxaban, but PT was prolonged only slightly (1.2-fold), at the respective ED_50_ values (Perzborn et al., 2005). These pharmacodynamic effects of rivaroxaban were mirrored by its antithrombotic activity. Rivaroxaban reduced thrombus formation in a dose-dependent manner after intravenous administration in the rat venous stasis model, and after oral administration in the rat and rabbit arteriovenous shunt models (Figure 2) (ED_50_ 0.1, 5.0, and 0.6 mg/kg, respectively), providing the first demonstration that rivaroxaban is effective in both arterial and venous thrombosis (Perzborn et al., 2005). In addition to prophylactic use, results obtained in an experimental rabbit jugular vein thrombosis model found that oral rivaroxaban (3.0 mg/kg) significantly reduced thrombus growth compared with oral control (36.9 ± 1.7% vs. 46.3 ± 1.3%; *p* < 0.05), demonstrating the potential of rivaroxaban to treat established thrombi (Biemond et al., 2007). No significant increase in bleeding time was observed at antithrombotic-effective doses (Perzborn et al., 2005; Biemond et al., 2007).

## Pharmacodynamic effect in clinical studies

### Phase I studies

Results of single- and multiple-dose escalation studies in healthy male subjects were consistent with those obtained in preclinical studies, confirming the anticoagulation effects of rivaroxaban in humans (Kubitza et al., 2005a,b). In the single-dose study, factor Xa activity was inhibited in a dose-dependent manner, and up to 75% inhibition was achieved with a single 80 mg dose of rivaroxaban (Kubitza et al., 2005a). Maximal factor Xa inhibition with suspension and tablet formulations was achieved after 45 min and 1–4 h, respectively (Kubitza et al., 2005a). Factor Xa activity was inhibited even at 24 h after administration at doses >5 mg (Kubitza et al., 2005a). Rivaroxaban prolonged the PT in a dose-dependent manner (assessed using Neoplastin Plus® from Roche Diagnostics, as in the preclinical studies). The inhibition of factor Xa activity and prolongation of PT both correlated strongly with plasma concentrations of rivaroxaban (*r* = 0.949 and *r* = 0.935, respectively) (Kubitza et al., 2005a).

After multiple dosing, maximal inhibition of factor Xa and prolongation of PT did not show cumulative effects, illustrating predictable pharmacodynamics with repeated dosing (Kubitza et al., 2005b).

In healthy male subjects, rivaroxaban (5 or 30 mg) dose-dependently inhibited TG in response to TF or collagen stimulation in platelet-rich and platelet-poor plasma, suggesting effective inhibition of TG induced by the extrinsic and intrinsic coagulation pathways (Graff et al., 2007, 2008). All parameters of ETP [area under the plasma concentration–time curve (AUC), peak, lag time] were significantly affected by rivaroxaban 5 and 30 mg doses, with the maximal effect achieved at 2 h (Graff et al., 2007, 2008). ETP peak (activated by collagen) remained reduced by 40% at 24 h after administration of a 30 mg dose (Graff et al., 2007, 2008); this was the first evidence to support the concept of once-daily administration (Graff et al., 2007, 2008).

### Phase II and III studies

In phase II studies investigating rivaroxaban compared with enoxaparin for the prevention of venous thromboembolism (VTE) after hip or knee replacement surgery, rivaroxaban exhibited predictable pharmacodynamics with both once- and twice-daily dosing (Turpie et al., 2006; Eriksson et al., 2007a). As was observed in phase I studies in healthy subjects, factor Xa inhibition and PT prolongation correlated closely with rivaroxaban plasma concentrations (Turpie et al., 2006; Eriksson et al., 2007b). The correlation of PT prolongation with rivaroxaban plasma concentration was also demonstrated in a phase II study, ODIXa-DVT, which investigated the optimal dose of rivaroxaban for the treatment of acute, proximal deep vein thrombosis (Agnelli et al., 2007; Mueck et al., 2007). These data demonstrated that the pharmacodynamic effects of rivaroxaban, as shown for PT prolongation, were consistent between the preclinical and clinical studies performed in healthy volunteers and patients, when the same assay methods were used (e.g., Neoplastin Plus® from Roche Diagnostics for PT) (Table 1). The consistent response among different patient populations and the low-to-moderate inter-individual variability in the exposure–PT relationship confirmed the predictable pharmacodynamics of rivaroxaban (Mueck et al., 2008, 2011). Furthermore, the observed pharmacodynamic effects in these early studies were successfully translated into clinical efficacy, as demonstrated in a number of large phase III studies in several thromboembolic indications, including the prevention of VTE after elective hip or knee replacement surgery (Eriksson et al., 2008; Kakkar et al., 2008; Lassen et al., 2008; Turpie et al., 2009), treatment of deep vein thrombosis and pulmonary embolism and prevention of recurrent VTE (The EINSTEIN Investigators, 2010; The EINSTEIN–PE Investigators, 2012), stroke prevention in patients with non-valvular atrial fibrillation (Patel et al., 2011), and secondary prevention in patients with acute coronary syndrome (Mega et al., 2012). In these phase III studies, rivaroxaban was shown to be superior or non-inferior to standard of care therapies (Table 2) with a similar or improved safety profile (including major bleeding events). The favorable benefit–risk profile of rivaroxaban was also evident across patient subgroups.

**Table 1:** **Prolongation of PT in preclinical and clinical studies of rivaroxaban**.

	**Study**	**Dose**	**PT prolongation**
			**(x-fold)**
*In vitro*	Platelet-poor human plasma (Perzborn et al., 2005)	0.30 (μg/L)	2.0
*Ex vivo*	Rat venous stasis model (Perzborn et al., 2005)	0.1 (mg/kg)^*^	1.8
	Rat arteriovenous shunt model (Perzborn et al., 2005)	5.0 (mg/kg)	3.2
	Rabbit arteriovenous shunt model (Perzborn et al., 2005)	0.6 (mg/kg)	1.2
	Rabbit jugular vein thrombosis (Biemond et al., 2007)	3.0 (mg/kg)^*^	1.5
		10.0 (mg/kg)^*^	1.8
Phase I	Single-dose escalation study in healthy subjects (Kubitza et al., 2005a)	10 mg	1.4
		20 mg	1.6
Phase II	VTE prevention after major orthopaedic surgery (ODIXa-HIP) (Eriksson et al., 2006)	10 mg once daily	1.4^†^

* After intravenous administration of rivaroxaban;

† Data on file. PT, prothrombin time; VTE, venous thromboembolism.

**Table 2:** **Clinical evaluation of rivaroxaban in phase III studies**.

**Study name**	**Clinical setting**	**Rivaroxaban dose regimen**	**Comparator**	**Efficacy outcome**
RECORD1 (Eriksson et al., 2008)	Thromboprophylaxis in patients undergoing total hip replacement surgery	10 mg od for 35 days post-surgery	Enoxaparin 40 mg od for 35 days post-surgery	Rivaroxaban was significantly more effective than enoxaparin for the prevention of VTE after total hip replacement surgery
RECORD2 (Kakkar et al., 2008)	Extended thromboprophylaxis in patients undergoing total hip replacement surgery	10 mg od for 31–39 days post-surgery	Enoxaparin 40 mg od for 10–14 days post-surgery	Extended thromboprophylaxis with rivaroxaban was significantly more effective than short-term enoxaparin for the prevention of VTE after total hip replacement surgery
RECORD3 (Lassen et al., 2008)	Thromboprophylaxis after total knee replacement surgery	10 mg od for 10–14 days post-surgery	Enoxaparin 40 mg od for 10–14 days post-surgery	Rivaroxaban was superior to enoxaparin for the prevention of VTE after total knee replacement surgery
RECORD4 (Turpie et al., 2009)	Thromboprophylaxis after total knee replacement surgery	10 mg od for 10–14 days post-surgery	Enoxaparin 30 mg bid for 10–14 days post-surgery	Rivaroxaban was superior to enoxaparin for the prevention of VTE after total knee replacement surgery
EINSTEIN DVT (The EINSTEIN Investigators, 2010)	Treatment of confirmed acute DVT without PE	15 mg bid for 3 weeks followed by 20 mg od for 3, 6, or 12 months	Enoxaparin 1 mg/kg overlapped and followed by warfarin or acenocoumarol (INR 2.0–3.0)	Rivaroxaban was non-inferior to standard therapy for the treatment of DVT and secondary prevention of VTE
EINSTEIN EXT (The EINSTEIN Investigators, 2010)	Treatment of confirmed symptomatic DVT or PE after 6–12 months' prior anticoagulation	20 mg od for 6 or 12 months	Placebo	Rivaroxaban was superior to placebo for the extended treatment of VTE
EINSTEIN PE (The EINSTEIN–PE Investigators, 2012)	Treatment of acute PE with or without DVT	15 mg bid for 3 weeks followed by 20 mg od for 3, 6, or 12 months	Enoxaparin 1 mg/kg overlapped and followed by warfarin or acenocoumarol (INR 2.0–3.0)	Rivaroxaban was non-inferior to standard therapy for the treatment of PE and secondary prevention of VTE
ROCKET AF (Patel et al., 2011)	Prevention of stroke or systemic embolism in patients with non-valvular AF	20 mg od (15 mg od in patients with CrCl 30–49 mL/min)	Warfarin [target INR of 2.5 (2.0–3.0)]	Rivaroxaban was non-inferior to warfarin for the prevention of stroke or systemic embolism in patients with non-valvular AF
ATLAS ACS 2 TIMI 51 (Mega et al., 2012)	Prevention of adverse cardiovascular outcomes in patients with a recent ACS	2.5 mg or 5 mg bid for 13–31 months	Placebo	At both doses, rivaroxaban reduced the risk of adverse cardiovascular outcomes after ACS alongside standard therapy

ACS, acute coronary syndrome; AF, atrial fibrillation; bid, twice daily; CrCl, creatinine clearance; DVT, deep vein thrombosis; INR, international normalized ratio; od, once daily; PE, pulmonary embolism; VTE, venous thromboembolism.

## Pharmacokinetic profile and clinical relevance

Preclinical animal studies have shown rivaroxaban to have predictable pharmacokinetic properties. Rivaroxaban had a rapid absorption, a moderate or high absolute bioavailability in rats (57–66%) and dogs (60–86%), and dose-proportional plasma concentrations with small variability between animals for the tested dose-interval (1.0–10.0 mg/kg in rats, 0.3–3.0 mg/kg in dogs) (Weinz et al., 2005). Rivaroxaban also showed a low plasma clearance rate, a moderate volume of distribution, and a short elimination half-life (Weinz et al., 2005). The tissue affinity of rivaroxaban was moderate, with only small or no retention observed; however, a species-dependent and fully reversible high plasma protein binding was observed (Weinz et al., 2005).

Rivaroxaban has a dual route of excretion through the biliary/fecal and urinary pathways, with a recovery of total radioactivity >91% being observed in all species tested. Urinary excretion of radioactivity was 25 and 52%, and fecal excretion was 67 and 43% of the dose in rats and dogs, respectively (Weinz et al., 2005). In humans, 36% of the administered dose was excreted renally as unchanged drug, whereas 28% of the dose was excreted in the feces (Weinz et al., 2009). Unchanged rivaroxaban was the main component in human plasma, with no major or active circulating metabolites present (Weinz et al., 2005, 2009; Bayer Pharma AG, 2013).

In phase I studies in healthy male subjects, rivaroxaban showed rapid absorption after oral administration and reached maximum plasma concentration (C_max_) within 4 h (Kubitza et al., 2005a,b). Exposure to rivaroxaban, in terms of the AUC and C_max_, was dose proportional for doses up to 10 mg irrespective of food intake, and dose proportional at all doses up to 30 mg twice-daily if administered with food (Kubitza et al., 2005a,b). No substantial accumulation of rivaroxaban at steady state was detected in the multiple-dose study (Kubitza et al., 2005b). The oral bioavailability of rivaroxaban was high (80–100%) for the 10 mg tablet dose, regardless of food intake. Under fasting conditions, absorption of a 20 mg tablet decreased to 66%, but approached completeness when the tablet was administered with food (Stampfuss et al., 2013). The terminal half-life of rivaroxaban ranged between 5 and 9 h in healthy young subjects and between ~11 and 13 h in elderly subjects (Kubitza et al., 2005a,b, 2008).

### Interactions with drugs affecting the cytochrome P450 3A4 and P-glycoprotein pathways

Rivaroxaban is metabolized via cytochrome P450 (CYP) 3A4, CYP2J2, and CYP-independent mechanisms (Bayer Pharma AG, 2013; Mueck et al., 2013). Based on *in vitro* investigations, it was shown that rivaroxaban is a substrate of the transporter proteins P-glycoprotein (P-gp) and breast cancer resistance protein. Consequently, drugs that affect CYP3A4, P-gp, or breast cancer resistance protein are expected to influence the pharmacokinetics of rivaroxaban (Gnoth et al., 2011; Mueck et al., 2013).

In phase I studies in healthy subjects, substrates of CYP3A4 or P-gp (such as the cardiac glycoside digoxin, the statin atorvastatin or the benzodiazepine midazolam) had no clinically relevant effect on the plasma pharmacokinetics of rivaroxaban (Kubitza et al., 2012b; Bayer Pharma AG, 2013; Mueck et al., 2013). Substances strongly inhibiting only one pathway of elimination (such as the antibiotics clarithromycin and erythromycin and the antifungal agent fluconazole) caused a modest increase in rivaroxaban exposure (54, 34, and 42% increases, respectively), but this did not result in clinically relevant increases in exposure that would necessitate dose adaptations (Bayer Pharma AG, 2013; Mueck et al., 2013). However, rivaroxaban exposure was found to be significantly increased when it was co-administered with strong inhibitors of both P-gp and CYP3A4, such as ketoconazole (158% increase in rivaroxaban exposure) and ritonavir (153% increase in rivaroxaban exposure) (Bayer Pharma AG, 2013; Mueck et al., 2013). The use of rivaroxaban is, therefore, not recommended in patients receiving systemic azole-antimycotics (such as ketoconazole, itraconazole, voriconazole, and posaconazole) or HIV protease inhibitors (such as ritonavir) (Bayer Pharma AG, 2013). Co-administration of the antibiotic rifampicin, a strong CYP3A4 inducer, led to an approximately 50% decrease in the AUC of rivaroxaban. It is likely that other strong CYP3A4 inducers (such as the anticonvulsants phenytoin, carbamazepine, and phenobarbital, and the herbal supplement St John's wort) may lead to reduced plasma concentrations of rivaroxaban (Bayer Pharma AG, 2013). These agents should, therefore, be co-administered with rivaroxaban with caution (Bayer Pharma AG, 2013).

### Effect of age, sex, body weight, and renal impairment on pharmacokinetic parameters

Phase I clinical studies have investigated the effect of age and sex on rivaroxaban pharmacokinetics. In healthy subjects aged >75 years, there was an increase in rivaroxaban exposure after the administration of a single 10 mg dose, as indicated by higher AUC values, compared with those observed in younger subjects (18–45 years), without a significant alteration in C_max_ (Kubitza et al., 2013). The AUC values were, on average, 41% greater than those observed in younger individuals (Kubitza et al., 2013). These changes were the result of reduced rivaroxaban clearance in elderly subjects, mainly owing to decreased renal function (Kubitza et al., 2013). Sex had no significant influence on the pharmacokinetics of rivaroxaban (Kubitza et al., 2013).

The influence of body weight on the pharmacokinetics of rivaroxaban was investigated in healthy subjects weighing ≤50 and >120 kg, compared with those with a normal body weight (70–80 kg) (Kubitza et al., 2007b). The C_max_ of rivaroxaban was unaffected in subjects >120 kg but was increased by 24% in subjects weighing ≤50 kg, with no effect on AUC. This was not considered clinically significant, suggesting that rivaroxaban is unlikely to require dose adjustment for body weight in adults (Kubitza et al., 2007b). Pharmacokinetic modeling has suggested that dose adjustment may be required in pediatric patients (Willmann et al., 2013); however, although studies are underway (clinicaltrials.gov, NCT01145859), no clinical data are yet available on the use of rivaroxaban in this population, and rivaroxaban is currently not recommended in patients aged <18 years (Bayer Pharma AG, 2013).

Rivaroxaban clearance is decreased with increasing renal impairment, leading to increased exposure. In subjects with mild (creatinine clearance [CrCl], 50–79 mL/min), moderate (CrCl, 30–49 mL/min), or severe (CrCl <30 mL/min) impairment of renal function, the AUCs of rivaroxaban were 44, 52, and 64% higher, respectively, compared with healthy control subjects after administration of a 10 mg rivaroxaban dose (Kubitza et al., 2010). The increase in AUC correlated inversely with CrCl (*r* = −0.45) (Kubitza et al., 2010). Rivaroxaban C_max_ was relatively unaffected, indicating that the increase in AUC was caused by reduced clearance and not by increased absorption (Kubitza et al., 2010). The influence of renal function on rivaroxaban clearance is considered to be moderate (Kubitza et al., 2010).

### Clinical relevance of pharmacokinetic parameters

The consistent, predictable pharmacokinetics and pharmacodynamics of rivaroxaban translated into predictable effects in the clinical programs (Table 2). Analysis of data obtained across pre-specified subgroups demonstrated consistent efficacy and safety outcomes irrespective of age or renal function (Eriksson et al., 2008; Kakkar et al., 2008; Lassen et al., 2008; Turpie et al., 2009; The EINSTEIN Investigators, 2010; Patel et al., 2011; Mega et al., 2012; The EINSTEIN–PE Investigators, 2012), including in patients with atrial fibrillation with renal impairment who received a reduced dose of rivaroxaban (15 mg instead of 20 mg) for prevention of stroke and systemic embolism (Patel et al., 2011). It should be noted that patients with CrCl <30 mL/min were excluded from all phase III studies; therefore, because limited data are available, rivaroxaban should be used with caution in patients with severe renal impairment (CrCl 15–29 mL/min) (Bayer Pharma AG, 2013). The use of rivaroxaban is not recommended in patients with CrCl <15 mL/min (Bayer Pharma AG, 2013).

Data from the subgroup analyses further confirmed that rivaroxaban dose adjustment is not required for age, sex, or body weight (Eriksson et al., 2008; Kakkar et al., 2008; Lassen et al., 2008; Turpie et al., 2009; The EINSTEIN Investigators, 2010; Patel et al., 2011; Mega et al., 2012; The EINSTEIN–PE Investigators, 2012).

## Pharmacodynamic interactions with drugs affecting hemostasis

Phase I studies in healthy male subjects evaluated the potential for interactions with commonly prescribed drugs that influence hemostasis, including non-steroidal anti-inflammatory drugs such as naproxen, platelet aggregation inhibitors such as clopidogrel, and the antiplatelet drug acetylsalicylic acid (ASA) (Kubitza et al., 2006, 2007a, 2012a; Bayer Pharma AG, 2013).

The maximum inhibition of factor Xa and prolongation of PT by rivaroxaban (administered as a single 15 mg dose) were unaffected in the presence of naproxen (500 mg) (Kubitza et al., 2007a). Rivaroxaban and naproxen given together significantly increased bleeding time compared with rivaroxaban alone (*p* = 0.017) (Kubitza et al., 2007a). However, this difference was small compared with the effect of naproxen given alone (Kubitza et al., 2007a).

Co-administration of clopidogrel (300 mg loading dose followed by 75 mg maintenance dose) and rivaroxaban (15 mg, given on day 2) had no effect on rivaroxaban-mediated inhibition of factor Xa or prolongation of PT (Kubitza et al., 2012a). Inhibition of adenosine 5′-diphosphate-stimulated platelet aggregation by clopidogrel was not affected by rivaroxaban (Kubitza et al., 2012a). Bleeding time was increased by clopidogrel, and co-administration of rivaroxaban and clopidogrel further increased bleeding time in a subset of subjects (Kubitza et al., 2012a).

Acetylsalicylic acid (500 mg loading dose followed by 100 mg maintenance dose) did not alter the effects of rivaroxaban (15 mg) on factor Xa activity or prolongation of PT (Kubitza et al., 2006). Bleeding time was significantly prolonged with ASA; the combination of rivaroxaban and ASA prolonged bleeding time slightly more than ASA alone (Kubitza et al., 2006).

These findings suggested a low potential for clinically relevant interactions of rivaroxaban with these commonly prescribed drugs; their use alongside rivaroxaban was investigated further in phase III studies.

In the phase III studies for the prevention of VTE after elective hip or knee replacement surgery (the RECORD program; Table 2), the concomitant use of ASA (no limit on dosage) and other platelet inhibitors with rivaroxaban (10 mg) was permitted (Eriksson et al., 2008, 2012; Kakkar et al., 2008; Lassen et al., 2008; Turpie et al., 2009). The relative rate ratios for any bleeding events, or major or non-major clinically relevant bleeding events, with different platelet inhibitors or ASA co-medication use vs. non-use remained relatively constant and were similar between the rivaroxaban and enoxaparin groups (Eriksson et al., 2012). In the EINSTEIN program (Table 2), ASA (up to 100 mg), and clopidogrel (75 mg per day), or both, were allowed if indicated (The EINSTEIN Investigators, 2010; The EINSTEIN–PE Investigators, 2012). Results obtained in a pooled analysis of patients in the EINSTEIN DVT and EINSTEIN PE studies indicated that co-administration of ASA with rivaroxaban increased the risk of bleeding, with no statistically significant increase in major bleeding, whereas use of non-steroidal anti-inflammatory drugs increased the overall risk of bleeding and the risk of major bleeding (Davidson et al., 2013). However, in general, an increased risk of bleeding complications is expected when combining an anticoagulant and antiplatelet agents, or other agents that affect hemostasis. In the ATLAS ACS 2 TIMI 51 study, increases in major bleeding (not related to coronary artery bypass grafting) and intracranial hemorrhage were observed when a low dose of rivaroxaban (2.5 or 5 mg twice daily) was co-administered with ASA and a thienopyridine (either clopidogrel or ticlopidine), but no increases in fatal bleeding were observed (Mega et al., 2012).

## Summary

Rivaroxaban is an oral, direct factor Xa inhibitor with high selectivity for factor Xa, and which has been shown in clotting assays in human plasma to effectively prolong PT, inhibit TG, and reduce both collagen- and TF-induced ETP. The antithrombotic potential of rivaroxaban was first demonstrated in *in vivo* preclinical studies in both arterial and venous thrombosis models, in which the potential for the treatment of established thrombi was also shown. These, together with data from initial (or early) clinical studies, provided further support that factor Xa is a viable target for anticoagulant therapy.

The pharmacodynamic and pharmacokinetic profiles of rivaroxaban seen in preclinical studies were further demonstrated in phase I and II studies in humans. Here, the rapid onset of action, short half-life, and plasma concentration-dependent effects on coagulation were confirmed. Data obtained during phase I and II studies also confirmed the predictability of rivaroxaban effects across a range of patient populations. Alongside the low potential for interacting with food or other drugs and the wide therapeutic window, the predictable pharmacokinetic and pharmacodynamic profile of rivaroxaban irrespective of age, sex, or body weight obviates the need for routine coagulation monitoring (in certain clinical situations laboratory testing may be helpful, such as prior to urgent surgery), which opens the possibility of offering fixed dosing to a broad range of patient populations.

The promising preclinical data, first confirmed by results of phase I studies, have been successfully translated into clinically meaningful results in patients. The efficacy and safety of rivaroxaban have been demonstrated in large-scale phase III studies in multiple indications, with rivaroxaban having non-inferior or superior efficacy to standards of care. In light of these data, rivaroxaban has been approved for the prevention or treatment of several thromboembolic indications. In order to ensure responsible use in patients, prescribers should adhere to the prescribing information for the respective indications. There are patient populations in which rivaroxaban is contraindicated or not recommended, including patients with severe renal impairment with CrCl <15 mL/min. With effective and safe anticoagulation coupled with oral, fixed dosing, rivaroxaban provides an attractive alternative to traditional anticoagulants and has the potential to simplify the management of thromboembolic disorders.

## Author contributions

Dagmar Kubitza designed, evaluated, and reported the clinical pharmacology studies referenced in this paper. She gave input by providing these data. In addition, she conceptualized the content of the manuscript. Elisabeth Perzborn was involved in the discovery of rivaroxaban and the characterization of the *in vitro* and *in vivo* pharmacological profile of rivaroxaban. She provided input by describing the preclinical pharmacodynamic profile. Scott D. Berkowitz contributed to the writing, revisions, and finalization of the manuscript. All authors revised the manuscript and approved it for submission.

### Conflict of interest statement

Dagmar Kubitza and Scott D. Berkowitz are employees of Bayer HealthCare Pharmaceuticals, Inc. Elisabeth Perzborn is a former employee of and consultant for Bayer HealthCare Pharmaceuticals, Inc., and has stock ownership in Bayer HealthCare Pharmaceuticals, Inc.
